# Dabigatran for Stroke Prevention in Nonvalvular Atrial Fibrillation: Answers to Challenging “Real-World” Questions

**DOI:** 10.1155/2012/867121

**Published:** 2012-05-07

**Authors:** Jorge Ferreira, Daniel Ferreira, Miguel Viana-Baptista, Paulo Bettencourt, Rui Cernadas, Francisco Crespo

**Affiliations:** ^1^Serviço de Cardiologia, Hospital de Santa Cruz, Avenida Prof. Reinaldo dos Santos, 2790-134 Carnaxide, Portugal; ^2^Serviço de Cardiologia, Hospital da Luz, Avenida Lusíada 100, 1500-650 Lisboa, Portugal; ^3^CEDOC, Faculdade de Ciências Médicas, Universidade Nova de Lisboa, Rua da Junqueira 126, 1349-019 Lisboa, Portugal; ^4^Serviço de Neurologia, Hospital Egas Moniz, 1349-019 Lisboa, Portugal; ^5^Serviço de Medicina Interna, Hospital de São João, Al. Prof. Hernâni Monteiro, 4200-319 Porto, Portugal; ^6^Unidade de Saúde Familiar da Aguda, Avenida Padre Guilherme 21, 4405-010 Arcozelo, Portugal; ^7^Hospital de Torres Vedras, Rua Dr. Ricardo Belo, 2560-324 Torres Vedras, Portugal

## Abstract

Dabigatran etexilate is a novel, oral, reversible, direct thrombin inhibitor that constitutes a major breakthrough for stroke prevention in patients with nonvalvular atrial fibrillation (AF). Dabigatran was the first new oral anticoagulant approved in Europe and became available in Portugal, for stroke prevention in nonvalvular AF, earlier than in most European countries. This paper is the joint effort of a panel of experts from different specialties and provides information on the use of dabigatran, in anticipation of the challenges that will come with increased usage.

## 1. Introduction

Dabigatran etexilate (designated as dabigatran from here onwards) and other new oral anticoagulants (OACs) constitute a major breakthrough for stroke prevention in patients with nonvalvular atrial fibrillation (AF). They have been shown to be an alternative to vitamin K antagonists (VKAs) that does not require routine laboratory control. Dabigatran (Pradaxa^®^, Boehringer Ingelheim, Ingelheim, Germany) is a reversible direct thrombin inhibitor and, in the RE-LY trial (Randomized Evaluation of Long-term anticoagulation therapY), a phase III study, 150 mg bid was more effective in terms of stroke prevention in nonvalvular AF than VKA, whereas 110 mg bid was as effective as VKA, with a lower risk of bleeding [1]. Soon after the completion of this trial, the US Food and Drug Administration (FDA) approved dabigatran 150 mg bid and 75 mg bid (for patients with a creatinine clearance of 15 to 30 mL/min) [2]. Subsequently, the European Medicines Agency approved the doses of 150 mg bid and 110 mg bid (for patients aged at least 80 years, with an elevated risk of bleeding or receiving verapamil) [3] as an alternative to warfarin for stroke and systemic embolism (SE) reduction in patients with nonvalvular AF. The American College of Chest Physicians (ACCP) Guidelines on Antithrombotic Therapy and Prevention of Thrombosis (9th ed) suggest dabigatran 150 mg twice daily rather than adjusted-dose VKA for patients with nonvalvular AF [4]. Moreover, recent quantitative benefit-harm and economic analyses in the UK support regulatory decisions that dabigatran offers a positive benefit to harm ratio when compared to warfarin [5].

Despite considerable variation among different countries, stroke has been appointed as the second leading cause of death worldwide [6]. Portugal was ranked the highest among Western European countries in terms of stroke mortality [7], and available data suggests that stroke not only continues to be the leading cause of death in this country [8], but also that its incidence is comparatively higher (crude annual incidence 3.05 and 2.69 per 1000 for rural and urban populations, resp.; corresponding rates adjusted to European standard population 2.02 and 1.73) [9]. There is no definite explanation for these facts, but a recent survey suggested that a higher prevalence of AF in the Portuguese population aged 40 and over, as compared to studies carried out in other countries, combined with an underutilization of VKAs, might contribute to these figures [10–12]. Furthermore, the Stroke and Atrial Fibrillation Ensemble (SAFE) II study suggested that, among patients with AF-associated stroke, underutilization of VKAs (70% of the patients had an indication for OAC, but less than 25% were anticoagulated) is frequently attributed to inappropriate reasons, such as fear of bleeding complications and low compliance [13].

Dabigatran was the first new oral anticoagulant approved in Europe for stroke prevention in nonvalvular AF and became available in Portugal earlier than in most other European countries. It seems reasonable to assume that its widespread utilization may soon become a reality, not only in hospital settings, but also in rural areas that lack laboratory facilities and particularly among clinicians previously reluctant in prescribing VKAs.

This paper is the joint effort of a panel of Portuguese experts from different specialties and provides information on the use of dabigatran, in anticipation of the challenges that will come with increased usage. The authors discuss the management of dabigatran-associated complications and the different clinical scenarios a clinician may encounter in patients treated with this new anticoagulant, underlining the paucity of systematic data, particularly on the management of recently reported hemorrhagic complications. Given the lack of specific clinical evidence, some of the recommendations below are based exclusively on the opinion of the authors and are identified as Author's Recommendations (AR).

## 2. Questions

### 2.1. Should the Decision to Start Treatment with Dabigatran or to Switch from VKAs to Dabigatran Be Restricted to Specialists Managing Patients with Atrial Fibrillation? What Is the Role of the General Practitioners (GPs)?

AF is frequent in the general population, its prevalence increases exponentially with age, and it is responsible for up to 20% of all strokes [14]. Due to the known limitations of VKAs, many patients with AF at a moderate-to-high risk of stroke, and with an indication for anticoagulation, are currently not treated [10]. For these patients dabigatran represents a new window of opportunity.

As first-line clinicians in national health systems, GPs can play a critical role not only in the diagnosis, but also in the treatment of AF. This position can be strengthened by the approval of new OACs, such as dabigatran, that has a better efficacy/safety profile and a broader therapeutic window [1].

The prerequisite for dabigatran prescription is not so much that the physician is a specialist or GP, but that he is competent in AF management and knowledgeable of its pharmacological treatment, dabigatran included.

When a GP diagnoses AF three issues must be addressed:

1. rate and rhythm management,
2. antithrombotic management,
3. diagnosis of concomitant cardiac disease and, when present, appropriate therapy [14].

In terms of antithrombotic therapy, recommendations for its use should be based on the presence (or absence) of risk factors and the risk of bleeding should also be taken into account [14]. According to the 2010 European Society of Cardiology (ESC) guidelines, the new CHA_2_DS_2_-VASc stroke risk stratification scheme may have a better predictive value when compared to the CHADS_2_ score [14]. As for the risk of bleeding, the HAS-BLED risk stratification score can be used for its determination [14]. With these results in mind and with the objective of obtaining a positive clinical benefit between prevention of thromboembolic events and avoidance of hemorrhagic complications, the decision to start OAC and to prescribe dabigatran or to switch from VKAs to dabigatran can be taken by any physician (AR).

### 2.2. How to Start Dabigatran? How to Switch from VKAs or Parenteral Anticoagulants to Dabigatran?

Before starting dabigatran, creatinine clearance (CrCl) must be evaluated in all patients using the Cockcroft-Gault formula [15, 16]. In Europe, dabigatran is contraindicated in patients with severe renal failure (CrCl < 30 mL/min) [17]. In the USA, the FDA approved dabigatran 75 mg bid for patients with a creatinine clearance of 15 to 30 mL/min [2]. A complete blood count should also be performed, since anemia and thrombocytopenia increase the risk of bleeding [4].

To switch from VKAs to dabigatran, physicians should first stop VKA and obtain an international normalized ratio (INR). If INR is below 2.0, dabigatran can be started immediately; if INR is between 2.0 and 3.0, dabigatran should be started in 2 days (AR). For patients with INR above 3.0, a further INR should be obtained after 2 days and dabigatran should be started as soon as the new INR is below 2.0, as stated above.

To switch from parenteral anticoagulants to dabigatran, with intermittent therapy (low molecular weight heparin), dabigatran should be given 0 to 2 hours before the time the next dose would be due. With continuous treatment (intravenous unfractionated heparin) dabigatran should be given at the time of discontinuation [17].

When switching from a VKA to dabigatran, clinicians should be aware that the benefits of dabigatran over warfarin are influenced by the efficiency with which the INR is controlled [18]. For all vascular events, nonhemorrhagic events and mortality, advantages of dabigatran over warfarin were greater at sites with poor INR control when compared to sites with good INR control. Nevertheless, the benefits of dabigatran 150 mg bid at reducing stroke or SE, dabigatran 110 mg bid at reducing major bleeding and both doses at reducing intracranial bleeding were consistent irrespective of the center's quality of INR control.

### 2.3. After Starting Dabigatran, When Should a Follow-Up Appointment Be Scheduled?

Dabigatran does not require routine laboratory monitoring. Nevertheless, it is important to monitor adherence to therapy and eventual adverse events. Patients should be provided with a phone contact for support.

The frequency of follow-up appointments in these patients must consider multiple factors: presence of comorbidities; previous treatment with VKAs; age and respective degree of autonomy; disease chronicity.

Patients should have a first visit within 3 to 6 months after starting dabigatran and thereafter according to their medical condition: in general, these patients should have biannual visits (AR). In elderly patients (>75 years) or patients with some degree of impaired renal function, quarterly visits are recommended (AR).

Since dabigatran is eliminated primarily in urine and since it is contraindicated in patients with severe renal failure [17], renal function should be assessed in the first appointment and every 6 months when mild-to-moderate renal dysfunction is present or whenever a decline of renal function is suspected.

### 2.4. How Can Adherence Be Checked and Improved?

The success of a clinical intervention depends not only on an accurate diagnosis and appropriate treatment, but also on the patient's commitment to comply with the prescribed therapy. Nonadherence is a major challenge for clinicians, especially in elderly patients, and, as with other chronic diseases (such as hypertension), AF patients should be monitored for compliance [19].

In view of the short half-life of dabigatran (12 to 14 hours after multiple doses) [3], no coagulation test can ascertain long-term compliance. As such, the evaluation of continued adherence must be clinically assessed.

To improve adherence, patient education should be reinforced. Prior to dabigatran prescription, clinicians should explain to patients and caregivers what an anticoagulant is; why it is being prescribed; what are its risks and benefits; what adverse events can occur and how to identify and deal with them. Patients and caregivers should be alerted of the importance of immediately contacting the physician in the event of bleeding or other adverse events and a special emphasis should be given to the risks of noncompliance. These measures should be complemented with educational brochures and easy access to nonscheduled appointments.

For reassurance, prescribing physicians should provide a phone contact to all patients starting dabigatran, especially those switching over from VKAs.

Other healthcare providers should be aware that the patient is anticoagulated, particularly if there is a possibility of surgery. In this respect, patient alert cards are useful tools, reinforcing patient adherence and providing other health professionals with information on the prescribing drug.

### 2.5. How to Deal with Dyspepsia?

Dyspepsia is a frequently reported adverse event with drugs commonly prescribed in primary care settings, such as NSAIDs or ticlopidine [20]. In the RE-LY trial, dyspepsia was observed in 11.3% and 11.8% of patients in the dabigatran arms and in 5.8% of patients in the warfarin arm [1]. Dabigatran-related gastrointestinal (GI) symptoms were most commonly of an esophageal/gastroesophageal reflux nature, typically occurring soon after drug initiation with a mild-to-moderate presentation and, in most cases, did not require drug discontinuation. Patients with GI symptoms had a higher risk of GI bleeding, irrespective of whether they were treated with dabigatran or warfarin [21], but the rate of GI bleeding was increased with dabigatran 150 mg bid versus warfarin (annual rate 1.51% versus 1.02%,*P* = 0.007) [1].

It is important to inform patients that dyspepsia may occur. The administration of dabigatran within meals (breakfast and dinner) can mitigate this effect [22]. The value of proton pump inhibitors in the management of dyspepsia, in this specific scenario, is not yet clear. However, it is known that a fifth of the patients enrolled in the RE-LY trial were on pantoprazole and although the bioavailability of dabigatran was reduced by about 12.5% in this subpopulation, a negative impact on clinical outcomes was not observed [17]. This data supports the use of pantoprazole for the management of dabigatran related dyspepsia.

### 2.6. In Which Patients Treated with Dabigatran Should Coagulation Be Monitored?

The RE-LY trial showed that dabigatran, when compared to warfarin, is an effective and safe treatment that obviates the need for routine laboratory monitoring.

The vast majority of patients will not benefit from routine and scheduled coagulation monitoring. In patients with an increased risk of bleeding, careful dose selection is advised. If available, the lower 110 mg bid dose should be used in patients aged at least 80 years; with high risk of bleeding; in patients with concomitant dabigatran and verapamil therapy [17]. In patients with moderate renal impairment, close clinical surveillance is recommended, including periodical assessment of renal function.

Only in exceptional situations may patients treated with dabigatran require anticoagulation status assessment. These situations are (1) cases of suspected overdose; (2) patients presenting to the emergency departments with acute bleeding, and (3) indication for urgent surgery (see also Section 2.7).

### 2.7. How Can the Anticoagulant Effect of Dabigatran Be Measured?

There is a close correlation between plasma dabigatran concentration and the degree of anticoagulant effect evaluated by the ecarin clotting time (ECT) and thrombin time (TT) [23]. To assess the risk of bleeding, qualitative tests such as activated partial prothrombin time (aPTT) or TT (other than the dabigatran calibrated Hemoclot thrombin inhibitor assay) may be used [24]. For a quantitative measurement of dabigatran plasma concentrations, only the dabigatran calibrated Hemoclot thrombin inhibitor assay (a diluted TT) is available [17].

Importantly, anticoagulant parameters depend on the time when the blood sample is taken relative to the time when the previous dose was given. A blood sample taken 2 hours after dabigatran ingestion (~peak level) will have different (higher) result in all clotting tests compared to a blood sample taken 10 to 16 hours (trough level) after ingestion of the same dose [25].

The following coagulation tests may be used to assess the risk of bleeding.

(i) aPTT: this test may be useful in determining an excess of anticoagulant activity, despite aPTT being less sensitive to the activity of dabigatran above therapeutic levels [23]. An aPTT >80 seconds (2 to 3 × baseline value) at trough is associated with a higher risk of bleeding [26]. A normal aPTT (not exceeding the upper limit of normal) indicates no clinically relevant anticoagulant effect of dabigatran. Most patients with therapeutic dabigatran plasma concentrations will have an aPTT ratio of about 1.5 to 2.0 (45 s to 65 s), at trough [23, 24]. However, as previously stated, aPTT should not be used as a quantitative measurement of dabigatran plasma concentrations.

(ii) TT: the actual test measure will depend on the coagulometer and the thrombin lot used for measurement.

(iii) Calibrated Hemoclot thrombin inhibitor assay (a diluted TT): an assay calibrated with dabigatran standards, to calculate dabigatran concentration [23]. In patients taking dabigatran for stroke prevention in nonvalvular AF, a diluted TT measure of >200 ng/mL of dabigatran plasma concentration (approximately >65 seconds) prior to the next drug intake (trough measure) is associated with a higher risk of bleeding [24]. A normal diluted TT measurement indicates no clinically relevant anticoagulant effect of dabigatran [26].

(iv) Prothrombin time and INR are unreliable in patients on dabigatran and false positive INR elevations have been reported [23, 27]. These tests should not be used (AR).

### 2.8. How Should Patients on Dabigatran with Major or Life-Threatening Bleeding Events Be Managed?

Even though life-threatening bleeding events are expected to be lower with dabigatran compared to VKA, it is advisable that hospitals develop specific protocols for emergency situations.

Presently, there is no specific antidote for dabigatran. However, it has recently been demonstrated that a specific humanized antibody fragment may provide a selective and rapid reversal of dabigatran activity. This antibody fragment is currently under clinical development for human use [28].

In the event of hemorrhagic complications, dabigatran must be discontinued and the source of bleeding investigated. As dabigatran is primarily excreted in the urine, adequate diuresis is needed and fluids should be administered as tolerated (AR). Transfusions with fresh frozen plasma, red blood cells, and fresh platelet concentrates may be considered, especially in cases where long-acting antiplatelet drugs have been used. Reversal agents like recombinant factor VIIa (rFVIIa) and activated or nonactivated prothrombin complex concentrates (PCCs) may also be considered, as suggested by experimental data [23, 29, 30]. Nevertheless, their efficacy remains unproven in the clinical setting. In a study with 12 healthy young volunteers who received dabigatran 150 mg bid for two and a half days, a single 50 IU/Kg bolus of PCC did not reverse the aPTT, ECT, and TT. However, no assessment of bleeding time or correlation with bleeding was performed [31].

When considering a surgical resolution for the life-threatening hemorrhagic complication, the risks of this procedure must be very carefully considered. If all of the above measures fail to control the bleeding, hemodialysis may be useful for dabigatran removal, especially in patients with renal impairment (see Section 2.11) [23, 32]. Hemoperfusion over a charcoal filter may also have some benefits [33].

As an example of an official emergency room protocol, the Pharmaceutical Management Agency of New Zealand recently issued the following guidelines for management of bleeding in patients treated with dabigatran [34].

- Mild bleeding: local hemostatic measures; delay next dose of dabigatran or discontinue treatment, as appropriate.
- Moderate-to-severe bleeding: local measures; fluid replacement; blood product transfusion; administration of antifibrinolytic agent for example, tranexamic acid IV (15–30 mg/kg)±continuous infusion (1 mg/kg/hr); consider Prothrombinex-VF 25–50 IU/kg, and repeat if necessary with hematology guidance.
- Life-threatening bleeding: previous measures; rFVIIa (100 mcg/kg by IV bolus), repeat if necessary with hematology guidance; hemodialysis, especially if renal failure is present.

### 2.9. How to Manage a Patient Treated with Dabigatran and Referred for Urgent Surgery?

Patients receiving antithrombotic therapy who undergo elective surgery or invasive procedures are at increased risk of bleeding. Therefore, these interventions may require temporary discontinuation of antithrombotic agents, and dabigatran is not an exception. Dabigatran should be discontinued 1 to 4 days prior to elective surgery, depending on the degree of renal impairment and risk of bleeding (Table 1) [17].

If the surgery or invasive procedure cannot be delayed to allow renal elimination of dabigatran and reversal of its anticoagulant effect, the degree of anticoagulation should be checked with the diluted TT assay (Hemoclot thrombin inhibitor assay) or aPTT [20, 26]. In the event of a normal diluted TT or aPTT (not exceeding the upper limit of normal) then no clinically relevant anticoagulant effect of dabigatran is present and surgery or invasive procedure can proceed without delay. If plasma concentration of dabigatran is >200 ng/mL or aPTT >80 seconds (2 to 3 × baseline value) and an urgent procedure with a high risk of bleeding is necessary, reversal of the anticoagulant effect of dabigatran with PCC or rFVIIa should be considered. If the blood sample is not collected at trough, reference values for coagulation tests are not currently available.

In the RE-LY study, patients receiving dabigatran were more likely to have a surgery or invasive procedure within 24 hours of withholding OAC and did so with a substantially lower risk of major bleeding than patients on warfarin (relative risk reduction of 82% for dabigatran 110 mg bid and 56% for 150 mg bid) [35].

When restarting dabigatran after surgery, clinicians should be aware of the rapid onset of its anticoagulant effect. Dabigatran can be resumed 24 to 72 hours after surgery, depending on the bleeding risk of the patient and procedure, as recommended for therapeutic heparin in the ACCP Guidelines [36].

### 2.10. Is a Patient Treated with Dabigatran Suitable for Neuraxial Anesthesia?

Neuraxial anesthesia comprises spinal (subdural space anesthesia) and epidural block (epidural space anesthesia) and is increasingly used in various types of surgery for pain relief, in the perioperative or postoperative periods [37].

The occurrence of spinal hematoma is a rare complication of neuraxial anesthesia, but may result in irreversible neurological damage and its risk is increased with antithrombotic therapy [38, 39]. Therefore, neuraxial anesthesia requires complete hemostatic function and dabigatran should be temporarily discontinued before the procedure. The timing of discontinuation should be adjusted in accordance to the patient's renal function and the procedure's risk of bleeding (Table 1).

Dabigatran may be initiated as soon as clinically indicated and at least 6 hours after the removal of epidural catheter [40].

### 2.11. Is Dialysis Efficient in Removing Dabigatran from Circulation?

Dabigatran is dialysable due to its relatively small molecular size and low plasma protein binding (~35%) [23]. This concept is supported by small studies performed in patients with a renal function ranging from normal to severely depressed. One open-label study demonstrated that the mean fraction of dabigatran removed by hemodialysis was 62% at 2 hours and 68% at 4 hours [41]. Another open-label study performed in patients with end-stage renal disease showed that the mean fraction of dabigatran cleared from plasma by high flow rate hemodialysis (400 mL/min) was 54 to 68% at 4 hours [32].

In emergency situations, hemodialysis can be employed to eliminate dabigatran and reduce its anticoagulant effect [23].

### 2.12. Can a Patient Treated with Dabigatran Receive Dual Antiplatelet Therapy (Aspirin Plus Clopidogrel)?

Aspirin and/or clopidogrel were used in 38.4% of patients during the RE-LY study and increased the relative risk of major bleeding by 60%, but the risk was lower with dabigatran 110 mg bid when compared to warfarin (HR 0.82, 95%CI: 0.67–1.00) [42]. Almost 5% of patients received dual antiplatelet therapy with aspirin and clopidogrel [43]. In these patients, the relative effects of both dabigatran 110 mg bid and 150 mg bid in comparison to warfarin in terms of major bleeding risk were consistent with the main trial results.

Adding aspirin to oral anticoagulation in patients with stable vascular disease (coronary, carotid, or peripheral arterial disease) does not reduce the risk of vascular events, including myocardial infarction [14]. As such, concomitant use of antiplatelets and oral anticoagulants should be cautious and reserved to special indications such as acute coronary syndromes or revascularization procedures. Dual antiplatelet therapy should be restricted to patients receiving coronary stenting. Implantation of drug eluting stents, which require a period of 6 to 12 months of dual antiplatelet therapy, should be avoided whenever possible [14]. The more potent new P2Y_12_ inhibitors, prasugrel, and ticagrelor, which have a higher bleeding risk than clopidogrel [44, 45], set new challenges to the safety of combining OAC with antiplatelet therapy.

In patients with nonvalvular atrial fibrillation and a clear recommendation for oral anticoagulation (CHA_2_DS_2_-VASc Score >1) and antiplatelet therapy, dabigatran is an advantageous alternative to warfarin, particularly with the 110 mg bid dose [42, 43].

Whenever dual or triple antithrombotic therapy is used, strict blood pressure control is strongly recommended [46].

### 2.13. How to Manage a Patient Treated with Dabigatran and Admitted for an Acute Coronary Syndrome? Which Antithrombotics Can Be Used If the Patient Is Referred for Primary Angioplasty? Can Dabigatran Be Administered Safely after an Acute Coronary Syndrome?

In the RE-LY study, there were specific recommendations for the management of acute coronary syndromes (ACSs). This issue was also tackled in the protocol of the RELY-ABLE trial, an extension of the dabigatran treated patients that completed the RE-LY trial (Table 2) [47].

ESC and ACC/AHA Guidelines recommend the use of dual antiplatelet therapy (aspirin plus P2Y_12_ inhibitor) in the setting of ACS [48–50] and this should also be applied to the patient treated with dabigatran. Since guidelines also recommend anticoagulation (heparin, fondaparinux, or bivalirudin) in the acute phase, it seems reasonable to temporarily discontinue dabigatran and start the recommended anticoagulants at least 12 hours after the last dose of dabigatran or as soon as the aPTT is <1.5 × baseline value.

If the patient is suitable for reperfusion therapy (ST-segment elevation myocardial infarction and less than 12 hours of symptoms) primary percutaneous coronary intervention (PCI) is preferable to fibrinolytic therapy. If primary PCI is not available, fibrinolysis should only be administered if aPTT is <1.5 × baseline value and potential gains outweigh the bleeding risks (AR).

Heparin should be administered during PCI according to the usual target of activated clotting time (ACT). There is a linear concentration-dependent increase in ACT with dabigatran plasma concentrations of up to 250 ng/mL [23].

Dabigatran can be restarted after the discontinuation of heparin, which generally occurs after coronary revascularization or after at least 48 hours of heparin therapy.

In the RE-LY trial, the annual rate of myocardial infarction (MI) was slightly higher in the dabigatran groups (0.82%/year for 110 mg and 0.81%/year for 150 mg) when compared with the warfarin group (0.64%/year) [51, 52]. This result may represent a play of chance or a more protective effect of warfarin against MI in patients with AF [53]. Nevertheless, in the RE-LY trial, this potential advantage of warfarin was exceeded by the benefit of dabigatran on cerebrovascular events, as reflected in the lower annual rate of vascular mortality observed in the dabigatran groups [1]. One meta-analysis of 7 trials found an increased risk of MI and ACS associated with dabigatran [54], but in clinical trials of primary prevention of venous thromboembolism, in patients undergoing major orthopedic surgery, no increase in ACS was detected in patients treated with dabigatran when compared to those treated with enoxaparin [55]. In the RE-LY platelet function substudy, no evidence of platelet activation was observed in the group of patients with AF receiving dabigatran when compared to warfarin [56].

### 2.14. How to Manage Anticoagulant Therapy in a Patient Treated with Dabigatran and Submitted to Percutaneous Ablation of AF?

In a recent study, administration of dabigatran to 123 consecutive patients after percutaneous ablation of AF was found to be well tolerated and safe in terms of bleeding complications and thromboembolic events, leading to the conclusion that dabigatran could be a good alternative to warfarin in this context [57].

In this study, periprocedural anticoagulation was based on a predetermined algorithm. Patients on dabigatran prior to AF ablation stopped this drug 36 hours before the procedure if estimated glomerular filtration rate (GFR) was >60 mL/min, 48 hours before the procedure if GFR was 40 to 60 mL/min, and 60 hours before the procedure if estimated GFR was <40 mL/min. Patients receiving dabigatran before ablation did not receive preprocedural enoxaparin.

Intraprocedural anticoagulation consisted on a weight-based bolus of unfractionated heparin administered when the transseptal sheath entered the left atrium, followed by a 1,000 units/hour infusion running through the transseptal sheath. Additional heparin administration depended on the ACT, targeted at 225 seconds. At the end of the procedure, heparin was reversed with protamine and all sheaths were removed.

After ablation patients received two doses of enoxaparin (0.5 mg/kg), the first one immediately at the end of the procedure and the second 12 hours later. Dabigatran was started 10 hours after the last enoxaparin dose (22 hours after the procedure) at a dose of 150 mg bid if the estimated GFR was >30 mL/min and 75 mg bid if the estimated GFR was between 15 and 30 mL/min (in Europe, the 75 mg bid dose is not approved for stroke prevention in patients with nonvalvular AF). Patients with a GFR <15 mL/min were not considered for dabigatran.

Dabigatran was maintained for at least 30 days after ablation. No postablation strokes, transient ischemic attacks, or systemic thromboembolic events were reported [57].

The Dabigatran for Peri Procedural Anticoagulation During Radiofrequency Ablation of Atrial Fibrillation (DAPPAR AF) trial, a prospective, open-label study, will start in a near future. In this trial, periprocedural anticoagulation will be managed in a similar way to that which is described in the previous study and this trial's primary outcome will be the incidence of periprocedural major bleeding complications [58].

### 2.15. How to Manage Cardioversion in a Patient Treated with Dabigatran?

Cardioversion to sinus rhythm (pharmacological or electrical) can be safely considered in AF patients treated with dabigatran. According to the ESC guidelines [14], in patients with AF of 48 h or longer duration (or AF of unknown duration) undergoing cardioversion, oral anticoagulants should be given for at least 3 weeks prior and 4 weeks after cardioversion.

This recommendation is also valid for dabigatran. Despite the fact that with dabigatran effective anticoagulation is obtained within 2 hours of administration, the possibility of a thrombus being already present in the left atrial appendage must be considered.

There is no prospective data on the safety of cardioversion under dabigatran treatment, but a post hoc analysis of the RE-LY trial revealed a low and comparable rate of cardioversion-related strokes in patients treated with dabigatran versus VKA [59]. In this study, the largest cardioversion study to date and the first evaluating dabigatran in this setting, more dabigatran patients underwent prior transesophageal echocardiography (TEE) (25.5% for dabigatran 110 mg, 24.1% for dabigatran 150 mg, and 13.3% for warfarin, *P* < 0.0001 for comparisons between dabigatran and warfarin) and although the prevalence of left atrial thrombus was not significantly higher in the dabigatran patients (1.8% for dabigatran 110 mg, 1.2% for dabigatran 150 mg, and 1.1% for warfarin, *P* = NS for comparisons between dabigatran and warfarin), it may still be prudent to recommended a TEE before a planned cardioversion in patients taking dabigatran.

It is important to point out that the routinely performed INR test, prior to cardioversion, is of no use in patients treated with dabigatran and noteworthy that the dabigatran-specific coagulation assay (diluted assay) will inform if the patient took dabigatran that particular morning, but gives no information on treatment compliance over the preceding 3 week period. Therefore, compliance of drug intake in the preceding 3 to 4 weeks should be specifically inquired and documented in the patient's file.

Finally, it is also important to recall that long-term anticoagulation is required for patients with a high thromboembolic risk, even if sinus rhythm is restored [14].

### 2.16. Is It Safe to Perform Thrombolysis for an Acute Ischemic Stroke in a Patient Treated with Dabigatran?

Despite the fact that there are no evidence-based data to answer this question, thrombolytic therapy is not recommended for anticoagulated patients with dabigatran [17]. There is only one reported case of thrombolytic treatment with intravenous rt-PA performed just below 4.5 h after the onset of symptoms and 7 hours after the last administration of dabigatran (aPTT 34.8 seconds, normal range 22.2–34.4 seconds; TT not reported) [60]. In this case, no hemorrhagic complications were observed. The Interventional Management Stroke (IMS) III trial proposed that administration of rt-PA or endovascular treatment could be considered 48 h after the last intake of dabigatran or within 48 h of last intake with a normal aPTT [61]. Future studies are needed in order to answer this question, until then the use of fibrinolytic agents for the treatment of acute ischemic stroke can probably be considered if the patient presents with a diluted TT or aPTT not exceeding the upper limit of normal, according to the local reference range, but careful evaluation of patient eligibility is strongly encouraged.

### 2.17. In a Patient with AF and an Acute Ischemic Stroke, or Transient Ischemic Attack, When Should Therapy with Dabigatran Be Initiated?

A substudy of the RE-LY trial showed that there was no increase in adverse events, including intracranial bleeding, with the two doses of dabigatran when compared to warfarin, between patients with and without previous stroke or transient ischemic attack [62]. Nevertheless, this trial excluded patients with transient ischemic attack or ischemic stroke in the two weeks before enrollment. Thus, while initiation of dabigatran seems safe after the first two weeks, further data on earlier initiation of dabigatran after stroke or transient ischemic attack is needed. However, it is important to underline that, in these settings, data concerning initiation of other anticoagulant drugs, including VKAs, is also lacking. An ongoing observational multicenter study, the Risk of early stroke recurrence in patients with Atrial Fibrillation (RAF) study, is currently evaluating timing of initiation of different anticoagulant drugs in patients with transient ischemic attack or acute ischemic stroke and AF (Paciaroni et al., unpublished data, 2011). Early introduction of dabigatran in transient ischemic attacks or minor ischemic stroke cases seems attractive, in view of its rapid anticoagulant action, and is probably feasible, but more data is needed in order to identify the optimal time for treatment initiation. Delaying introduction of anticoagulant therapy in patients with extensive ischemic lesions, as proposed by current guidelines [63], should also be advocated in the case of dabigatran, to avoid hemorrhagic transformation.

### 2.18. How Should Intracranial Bleeding Be Managed in a Patient Treated with Dabigatran?

Fewer intracranial hemorrhagic events are expected in patients under treatment with dabigatran than in patients treated with warfarin. In the RE-LY trial, in comparison with warfarin treatment, there was a marked reduction of intracranial bleeding with both doses of dabigatran (69% reduction with dabigatran 110 mg bid and 60% reduction with dabigatran 150 mg bid, *P* < 0.001 for comparisons between dabigatran and warfarin) [1]. Nevertheless, it is important to understand how to manage these situations in case they do occur.

As there is no antidote for dabigatran, currently available, supportive treatment in order to stop bleeding is the mainstay of management of intracranial hemorrhagic events. Dabigatran should be stopped immediately and symptomatic treatment should be initiated. Further attitudes should be individualized according to the severity of the hemorrhage [64, 65], and measures described in Section 2.8 should be taken into account.

## 3. Conclusion

The questions answered in this paper represent some of the relevant issues clinicians will face when asked to manage nonvalvular AF patients treated with dabigatran. Information about these and other management issues should be easily and widely available, in order to improve correct patient and drug dosage selection, taking into account variables such as age, renal function, and comorbidities.

“Real-world” experience with dabigatran and further evidence from ongoing clinical trials will dictate the need for adjustments to the recommendations in this paper.

## Figures and Tables

**Table 1:** Guide to the discontinuation of dabigatran before elective surgery or invasive procedure [17].

Renal function	When to stop dabigatran before surgery	
(CrCl mL/min)	Standard risk of bleeding	High risk of bleeding
>80	24 hours before	2 days before
>50 and ≤80	1-2 days before	2-3 days before
>30 and ≤50	2-3 days before	4 days before

**Table 2:** Recommendations for the management of acute coronary syndromes in the RE-LY and RELY-ABLE studies [47].

(1) Dabigatran should be temporarily discontinued
(2) Aspirin, clopidogrel and glycoprotein IIb/IIIa inhibitors may be administered according to usual clinical practice
(3) Heparin can be started 12 or more hours after the last dose of dabigatran or as soon as the aPTT is <1.5 × baseline value
(4) Reperfusion with primary PCI is preferable to fibrinolysis
(5) During PCI the ACT should be measured and heparin administered as needed according to usual practice
